# Identification of a Novel Walnut Iron Chelating Peptide with Potential High Antioxidant Activity and Analysis of Its Possible Binding Sites

**DOI:** 10.3390/foods12010226

**Published:** 2023-01-03

**Authors:** Chaozhong Fan, Xintong Wang, Xiwang Song, Ronghao Sun, Rui Liu, Wenjie Sui, Yan Jin, Tao Wu, Min Zhang

**Affiliations:** 1 State Key Laboratory of Food Nutrition and Safety, Food Biotechnology Engineering Research Center of Ministry of Education, College of Food Science and Engineering, Tianjin University of Science & Technology, Tianjin 300457, China; 2 China-Russia Agricultural Processing Joint Laboratory, Tianjin Agricultural University, Tianjin 300384, China

**Keywords:** iron-chelating peptides, walnut peptides, structural characteristics, antioxidant activity, iron fortified food

## Abstract

Peptide iron chelate is widely regarded as one of the best iron supplements for relieving iron deficiency. In this study, a new type of walnut peptide iron (WP-Fe) chelate was prepared using low molecular weight walnut peptides (WP) as raw materials. Under the conditions of this study, the chelation rate and iron content of the WP-Fe chelate were 71.87 ± 1.60% and 113.11 ± 2.52 mg/g, respectively. Fourier transform infrared spectroscopy (FTIR), zeta potential, amino acid composition, and other structural analysis showed that WP-Fe is formed by the combination of carboxyl, amino and carbonyl with Fe^2+^. The WP-Fe chelate exhibits a honeycomb-like bulk structure different from that of WP. In addition, we predicted and established the binding model of ferrous ion and WP by molecular docking technology. After chelation, the free radical scavenging ability of the WP-Fe chelate was significantly higher than that of the WP. Overall, the WP-Fe chelate has high iron-binding capacity and antioxidant activity. We believe that peptides from different sources also have better iron binding capacity, and peptide iron chelates are expected to become a promising source of iron supplement and antioxidant activities.

## 1. Introduction

Iron is an indispensable trace mineral for almost all living things, which has an important impact on physiological activities such as oxygen transport, maintenance of normal hematopoietic function, and immune skills of the body [1]. Iron deficiency (ID) in the human body is prone to iron deficiency anemia, and even increases the risk of diseases such as neurodegenerative diseases [2], chronic kidney disease [3], and chronic heart failure [4]. In addition, it has been reported that iron, zinc, copper, and other trace elements have an important relationship with coronavirus infection (COVID-19), since they are important metalloproteins that bind to proteins associated with virus infection [5,6]. There are two main forms of iron deficiency: absolute iron deficiency and functional iron deficiency. Absolute iron deficiency occurs when iron stores are insufficient to meet the individual needs, and is especially common in young children (under 5 years of age) and premenopausal (especially pregnant) women. Functional iron deficiency is iron-deficient erythropoiesis and anemia due to the retention of iron from plasma despite adequate iron stores in the body. Absolute defects and functional defects can coexist [3]. The management of ID is an important and complex challenge facing medical, nutrition, and public health practitioners worldwide [7].

Intravenous iron supplementation has been around for nearly a decade and has shown promising improvement in patients with heart failure and iron deficiency, but the long-term treatment and safety data are unclear [4]. Oral iron supplementation is often recommended as the preferred treatment method, however, various potential barriers, mainly due to low bioavailability, severe side effects, and high cost, still need to be addressed [3,7]. Traditional iron supplements are mainly in the form of inorganic salts. When the human intestine absorbs iron, it is easily affected by phytic acid and phosphoric acid, resulting in its bioavailability being less than 15% [8,9]. Therefore, it is necessary to develop an ideal iron supplement to alleviate the adverse symptoms caused by iron deficiency.

In recent years, a large number of research reports have found that some bioactive polypeptides extracted from food proteins interact with ferrous ions to form polypeptide iron complexes including barley protein peptides [10], the effect of duck egg white peptide [11], oat peptides [12], mung bean polypeptides [13], hairtail peptides [14], etc., which to some extent improve the iron absorption, bioavailability, and solubility. Qu et al. [15] prepared corn peptide iron chelate with iron supplement and ACE inhibitory activity from corn. It may have the functions of ferrous ion and active polypeptide at the same time, which needs to be further studied. On one hand, polypeptide–iron complexes can improve the bioavailability of ferrous ions through polypeptide channels and promote the multi-channel absorption of iron. On the other hand, the adverse effects of phytic acid and fiber on the utilization of free iron ions can be effectively avoided through the presence of metal chelating peptides. Multiple reports have demonstrated improved in vivo iron bioavailability by peptide–ferrous complexes [11,14]. In addition, the peptide–iron complex may have the biological activity of the peptide while improving the bioavailability of iron [15,16].

Walnut meal is considered a by-product of walnut oil processing. Studies have shown that walnut meal contains a lot of high-quality protein, but it is usually used as animal feed or discarded, resulting in a waste of resources [17]. In order to improve the comprehensive utilization value of walnut and promote the development of ecological greening, we prepared a new type of iron chelating peptide using a low molecular weight walnut peptide. We verified the chelating effect and action site of low molecular weight walnut peptides by techniques such as Fourier transform infrared spectroscopy, particle size distribution, zeta potential, scanning electron microscopy, elemental composition analysis, amino acid composition, and peptide chain identification. In addition, we also used molecular docking technology to establish the binding model of peptides and ferrous ions. This provides a theoretical basis for the development of functional foods to promote iron absorption and prevent iron deficiency and iron deficiency anemia.

## 2. Materials and Methods

### 2.1. Materials and Reagents

Walnut polypeptides were purchased from Tianjin Peptide Valley Biotechnology Co. Ltd, (Tianjin, China). FeSO_4_·7H_2_O, anhydrous ethanol, 2,2-diphenyl-1-picrylhydrazyl (DPPH), and other chemical reagents were all of analytical grade and were purchased from Tianjin Sinopharm Chemical Reagent Co. Ltd., (Tianjin, China). The water used in the experiment was deionized water.

### 2.2. Preparation of WP-Fe Chelate

Walnut polypeptides were prepared from walnut meal by enzymatic hydrolysis from our cooperative company. Walnut meal was pretreated to make a homogenate and hydrolyzed with alkaline protease. Enzymatic hydrolysis conditions: substrate concentration 5%, enzyme amount 5000 U/g, pH 8.0, enzymatic hydrolysis time 5 h. The enzymolysis solution was boiled to kill the enzyme, and then centrifuged by 5000× *g* to take the supernatant. The obtained supernatant solution was freeze-dried to obtain the walnut polypeptide (WP).

The preparation method of WP-Fe was adapted from Zhang et al. [18] with slight modifications. Walnut polypeptide was ultrafiltrated through a 3 kDa filter membrane and made into a 30 mg/mL WP solution. Then, FeSO_4_·7H_2_O was added to WP solution with a peptide–iron ratio of 3:1 (*w*/*w*), and the pH value of the solution was adjusted to 4.5. The reaction was carried out in an oscillating water bath incubator at 45 °C for 60 min. After the chelation reaction, the reaction solution was mixed with four times the volume of absolute ethanol and left for 120 min. The precipitate was collected after centrifugation at 8000× *g* for 15 min and dried in a vacuum freeze dryer. Samples were stored at −20 °C.

### 2.3. Determination of Iron Content and Complex Yield

The content of ferrous ions in the WP-Fe chelate was determined by the method reported by [19] with slight modifications. A total of 0.2 mL of the sample solution was accurately weighed and mixed with 1 mL of 1 moL/L hydrochloric acid, 0.2 mL of 100 mg/mL hydroxylamine hydrochloride solution, and 1 mL of acetic acid–sodium acetate buffer solution, and left at room temperature for more than 30 min. Then, 0.2 mL of 1 mg/mL o-phenanthroline solution was added to the container, shaken to constant volume, and the absorbance was measured at a wavelength of 510 nm with distilled water as the reference solution. The following equations were used to calculate the iron content and chelate yield of the samples.

Iron content (mg/g) = m_2/_m_0_

Chelating rate (%) = (m_2_/m_1_) × 100%

where m_1_ represents the content of iron before chelation (mg); m_2_ represents the content of iron in WP–Fe chelate (mg); m_0_ represents the mass of WP-Fe chelate (g).

### 2.4. Measurement of FTIR

A total of 1 mg of the WP or WP-Fe complex was mixed with 200 mg of dry KBr, ground, and compressed into discs. The structures of the WP and WP-Fe complexes were then characterized by a Fourier transform infrared spectrometer IS50 (Nicolet, USA) instrument in the wavenumber range of 400–4000 cm^−1^.

### 2.5. Scanning Electron Microscopy (SEM) and Energy Dispersion (EDS) Measurements

Measurements: A small amount of the WP and WP-Fe powders was attached to an aluminum post of a scanning electron microscope device (S-3400N, Hitachi, Japan) via conductive glue. After the samples were sprayed with gold, the samples were observed and photographed by scanning electron microscope at magnifications of 500 times, 1000 times, and 5000 times, respectively. The elemental compositions of the WP and WP-Fe chelate were detected by point-scanning with an energy spectrometer.

### 2.6. Determination of Particle Size Distribution and Zeta Potential

The freeze-dried samples of WP and WP-Fe were folded into 1 mg/mL solutions, respectively, and passed through a 0.22-micron filter. The sample particle size distribution and zeta potential were measured with a particle size laser.

### 2.7. Determination of Amino Acid Composition

A total of 80 mg of ultrasonically treated WP and WP-Fe powders were put into 10 mL of 6 mol/L hydrochloric acid containing 0.1% (*w*/*v*) phenol, hydrolyzed at 110 °C for 22 h, and the hydrolyzed solution was filtered with qualitative filter paper and diluted to 50 mL. Subsequently, 1 mL of the diluted hydrolyzate was concentrated twice with a vacuum centrifugal concentrator (5430R, Eppendorf, Germany) at 48 °C and dissolved in 1 mL of distilled water. The sample solution was filtered through a 0.45 μm filter, and the amino acid composition was determined using an amino acid analyzer (S-433(D), Sykam, Germany).

### 2.8. Identification of Peptide Chains

The liquid A used in the liquid phase was a 0.1% formic acid aqueous solution, and the B liquid was a 0.1% formic acid acetonitrile aqueous solution (acetonitrile is 84%). A liquid chromatography column (0.15 mm * 150 mm, RP-C18, Column Technology Inc., Fremont, CA, USA) was equilibrated with 95% solution A, and the sample was loaded by an autosampler onto Zorbax 300SB-C18 peptide traps (Agilent Technologies, Wilmington, DE, USA), and then separated by a liquid chromatography column. The relevant liquid phase gradient settings were as follows: 0–50 min, the linear gradient of liquid B was from 4% to 50%; 50–54 min, the linear gradient of liquid B was from 50% to 100%; 54–60 min, solution B was maintained at 100%. The enzymatic hydrolysis products were separated by capillary high performance liquid chromatography and then analyzed by a Q Exactive mass spectrometer (Thermo Fisher). Analysis time: 60 min. The original file of the mass spectrometry test (raw file) was searched with the software MaxQuant 1.5.5.1 to the corresponding database, and finally the peptide chain sequence was obtained.

### 2.9. Determination of DPPH Free Radical Scavenging Ability

We prepared a 0.2 mmol/L DPPH solution with absolute ethanol, mixed a sample of a certain concentration with 0.2 mmol/L DPPH in a volume ratio of 1:1, and reacted at room temperature for 30 min in the dark, denoted as A_1_; absolute ethanol and an equal amount of sample was mixed, recorded as A_2_; and anhydrous ethanol mixed with an equal amount of 0.2 mmol/L DPPH, recorded as A_0_. Each group was measured three times and averaged. The DPPH free radical scavenging rate was calculated as follows.

$$\mathrm{Activity}\;\mathrm{of}\;\mathrm{scavenging}\;\mathrm{DPPH}\;\mathrm{radicals}\;(\%)=[1–\;(A_{2}\;-\;A_{1})/A_{0}]\;\times \;100\%$$

where (*A*_2_ − *A*_1__)_ is the absorbance of the sample, *A*_0_ is the absorbance of the control.

### 2.10. Determination of Hydroxyl Free Radical Scavenging Ability

The hydroxyl scavenging ability was determined by the Fenton reaction-salicylic acid method. The sample was composed of 9 mmol/L salicylic acid-ethanol solution, 9 mmol/L ferrous sulfate solution, and 8.8 mmol/L hydrogen peroxide solution, and 1 mL of each was added to the test tube sequentially. The reaction was carried out at 37 °C for 30 min, and the absorbance was measured at 510 nm.

### 2.11. Molecular Docking

The molecular docking of the walnut peptide and ferrous ion was carried out using AutoDock Vina 1.1.2 software. First, we constructed the structure of the ferrous ion and the peptide chain. The Fe^2+^ structure was obtained from PUBCHEM. The structures of the short peptides were built using the Maestro 13.0 academic version. In addition, ADFRsuite 1.02 was used to convert all processed iron ions and short peptide structures into the PDBQT format necessary for AutoDock Vina 1.1.2 docking. The second step was to set up the docking box. The third step was to set the parameters. The verbosity level was set to 32, and the other parameters remained in the default settings. The highest score among the output docked conformations was the conformation that we considered ideal. Finally, PyMol 2.5 was used to optimize the 3D effect of the docking results.

### 2.12. Data Analysis

Excel 2019 and Origin 2017 were used to process and graph the data, and SPSS 22.0 was used to analyze the significance of the data.

## 3. Results and Discussion

### 3.1. Iron Chelation Rate and Iron Content

The WP-Fe chelate was obtained under the conditions of a material ratio of 3:1, temperature of 45 °C, pH of solution 4.5, and rotation speed of 160 g. The iron content of the WP-Fe chelate obtained under this condition was 113.11 ± 2.52 mg/g, and the chelation rate was 71.87 ± 1.60%. This proves that the low molecular weight walnut peptides have a positive contribution to ferrous ion chelation. Zhang et al. [18] extracted mung bean polypeptide from mung bean and had a good binding effect with ferrous ion. Qu et al. [15] obtained corn ACE inhibitory peptide–ferrous chelate by ultrasonic-assisted enzymatic hydrolysis, and its ferrous content was 66.39 ± 1.49%. In addition, it has been studied that the chelating ability of metal ions may be related to the type of polypeptide, the content of acidic amino acids, and the relative molecular mass. Low molecular weight peptides may favor metal ion binding.

### 3.2. FTIR

**Figure 1:**
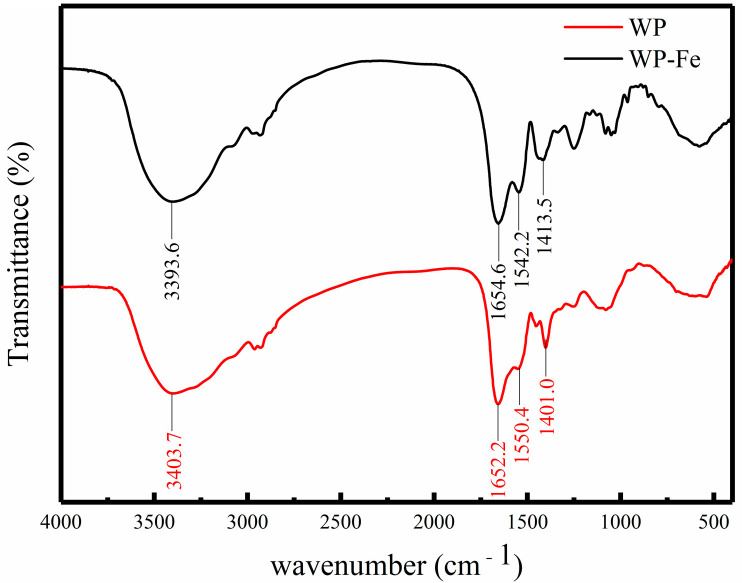
FTIR spectra of the WP and WP-Fe chelate.

The change in the FTIR absorption peak can reflect the interaction between the ferrous ions and the organic group of the WP. We found that the absorption peaks of the WP and WP-Fe chelate were significantly different (Figure 1). At 3404 cm^−1^ was the strong absorption peak of -NH_2_. When SCP bound to iron ions, its strong absorption peak changed from 3404 cm^−1^ to 3394 cm^−1^. This result may be caused by the transition from the N-H bond to the Fe-N bond. The absorption band of WP at 1652 cm^−1^ was characterized as an amide I band. After binding with iron, the band corresponding to the amide I group moved to 1655 cm^−1^, which indicated that the C=O group may combine with Fe^2+^ to form C-O-Fe. Similarly, the characteristic peak of -COOH moved from 1401 cm^−1^ to 1414 cm^−1^ with the chelation reaction, which was directly correlated to the structure of -COO-Fe. All in all, after the walnut polypeptide was chelated with ferrous iron, the position of its characteristic absorption peak and the relative absorption intensity changed significantly, indicating that the polypeptide–ferrous chelate is a new substance unlike the polypeptide.

It can be seen from the above reasoning that the carboxyl, carbonyl, and amino groups in walnut peptides play an important role in the process of chelating ferrous ions, and ionic bonds and coordination bonds may be their main modes of action. Similar conclusions were also reported for many different types of peptides. In the corn ACE inhibitory peptide–ferrous chelate and whey peptide–calcium chelate, metal ions were mainly combined with the carboxyl group, carbonyl group, and amino group of the polypeptide [15,20].

### 3.3. Particle Size and Zeta Potential Analysis

**Figure 2:**
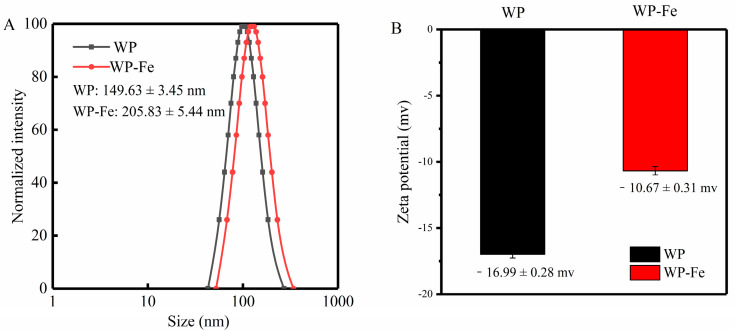
Particle size distribution (**A**) and zeta potential (**B**) of the WP and WP-Fe chelate.

The particle size distribution curve is an important physical parameter for judging composite materials. The particle size distributions of WP and WP-Fe are shown in Figure 2A. It can be found in the figure that compared with the average particle size of WP of 149.63 ± 3.45 nm, the particle size range was 42.85–270.65 nm, the average particle size of the WP-Fe chelate was significantly increased (205.83 ± 5.44 nm), and the particle size range was 52.24–340.21 nm. The results showed that after the iron chelation reaction, the particle size of WP-Fe became larger. On one hand, this is because the ferrous ions change the spatial structure of the peptide chain. On the other hand, the addition of ferrous ions causes aggregation between peptide chains, that is, one ferrous ion binds two or more peptide chains at the same time, which is also the main reason for the increase in the particle size of the peptide chains. This is consistent with the results reported by Tian et al. [21], where a ferrous ion can bind to one or more peptide chains simultaneously. In addition, the polydispersity index (PDI) of the WP and WP-Fe chelate were 0.165 and 0.341, respectively, indicating a uniform and stable particle size distribution.

The zeta potential can reflect the surface charge state of peptides and chelates, which is helpful for our subsequent analysis of their direct mechanism of action. The results are shown in Figure 2B; compared with WP, the zeta potential of the WP-Fe chelate increased significantly from −16.99 ± 0.28 mv to −10.67 ± 0.31 mv (*p* < 0.05). The increase in charge proves that the ferrous ions neutralize the negative charge on the surface of the polypeptide, and it also proves that there is an electrostatic interaction in the chelation reaction. The same results were also confirmed by a new Antarctic krill zinc chelate developed by Sun et al. [22].

### 3.4. SEM and EDS Analysis

**Figure 3:**
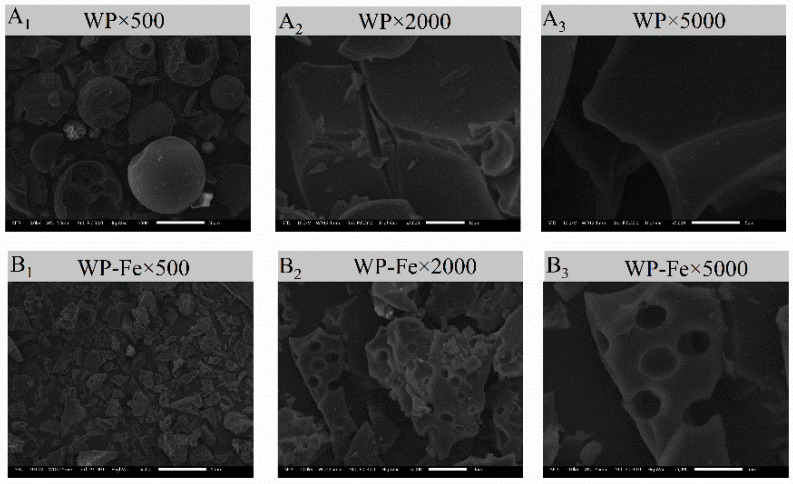
Microstructure of the WP (**A**) and WP-Fe chelate (**B**) determined by SEM.

The microscopic surface structures of WP and WP-Fe were observed by scanning electron microscopy at a 500×, 2000×, and 5000× field of view. As shown in Figure 3A, WP presents a smooth lamellar or spherical structure, while the WP-Fe surface shown in Figure 3B showed a block-like structure with honeycomb-like pores on the surface. The main reason for the difference between WP and WP-Fe may be that ferrous ions destroy the structure of the original polypeptide, which makes the recombination between the peptide chain to form the compact result of WP-Fe. This result is consistent with our particle size results. The ferrous ions form a cyclic structure by combining with the functional group of the polypeptide, and the aggregation between the peptide chains, which makes the surface of the WP-Fe chelate present many porous structures [23]. Similarly, a new type of sea cucumber–iron chelating peptide was prepared in our previous study, which was also significantly different from the surface structure of the peptide [24].

**Figure 4:**
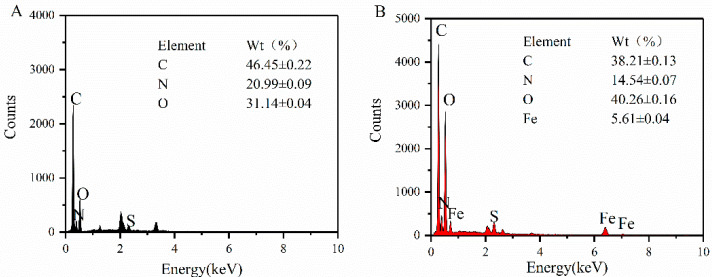
Elemental composition analysis of the WP (**A**) and WP-Fe chelate (**B**).

The surface element composition of the WP and WP-Fe chelate was analyzed by EDS, and the results are shown in Figure 4A,B. WP only contains C (40.87%), N (10.77%), and O (18.92%), while the elemental composition of the WP-Fe chelate was C (32.45%), N (8.68%), O (29.77%), and Zn (13.63%). In addition, the WP-Fe chelate had two iron peaks that were completely different from WP. Compared with WP, the signal intensity and iron content of the WP–Fe chelate were significantly enhanced. This finding suggests that iron is successfully chelated with WP after the reaction. With the same results, Zhang et al. [25] used EDS to identify the successful coupling of oyster peptides with zinc.

### 3.5. Amino Acid Composition

The results of the infrared spectroscopy and zeta potential have shown that the types of amino acid functional groups are closely related to the binding of ferrous ions. In order to better analyze the binding site of ferrous ions, we detected the amino acid content of WP and WP-Fe (Table 1). We found that Glu and Arg were the most abundant in walnut, accounting for 32.79% of the total amino acids. After chelation with Fe^2+^, the proportion of Glu and Arg in the WP-Fe chelate increased by 3% and 5%, respectively, which may be because the side chain groups provided by Glu and Arg play an important role in the ferrous ion chelation effect. For example, carboxyl groups provide binding sites for metal ions through electrostatic interactions or coordination [26,27,28]. Similar results were also found in the tilapia skin collagen peptide zinc chelate developed by Meng et al. [29]: the acidic and basic amino acids in the peptide chelate zinc ions through electrostatic or coordinative interactions.

### 3.6. Polypeptide Sequence of WP-Fe Chelate

The amino acid sequence of WP-Fe was determined by LC-MS/MS and 27 polypeptides were identified (Table 2), which had not appeared in other studies. The molecular weights of these peptide chains were mainly distributed between 1000 and 2000 Da, which indicates that the walnut peptides in this range have strong iron-binding ability. Thus far, both high molecular weight peptides and low molecular weight peptides have been reported to have good metal chelating ability. Sun et al. [30] concluded that the components with molecular weights greater than 3000 Da in the polypeptide were negatively correlated with the iron-binding capacity. Interestingly, Mahoney [31] found that iron mainly binds to chicken muscle protein peptides with a molecular weight greater than 10 kDa. The score of the peptide indicates the credibility of the existence of the amino acid sequence, of which the two peptide chains of GEHIEESR and HAVSEGTK had the highest credibility. It is worth noting that 23 of these peptide chains contained at least one glutamic acid or arginine. This again proves that the binding sites of ferrous ions are mainly the side chain groups of glutamic acid and arginine, which is consistent with the analysis of the amino acid composition. In addition, we found that the way arginine appears in these peptide chains is always at the N-terminus of the peptide chain, which may be because the arginine at the N-terminus is beneficial to donate a lone pair of electrons for ferrous ions. Ferrous ions combine with the lone pair of electrons provided by arginine to combine peptide chains with peptide chains, which is why the particle size of the WP-Fe chelate increases [32].

### 3.7. Molecular Docking

Molecular docking techniques are commonly used to study molecular forces between proteins and metal ions and to predict their binding models [33,34]. In this paper, the interaction between ferrous ions and low molecular weight walnut polypeptides was docked by AutoDock Vina 1.1.2 software. The docking results of ferrous ions and peptides are shown in Table 3, which confirmed that the carboxyl group provided by the acidic amino acid plays an important role in the binding process, and the binding distance was between 2.0 and 2.8 angstroms.

**Figure 5:**
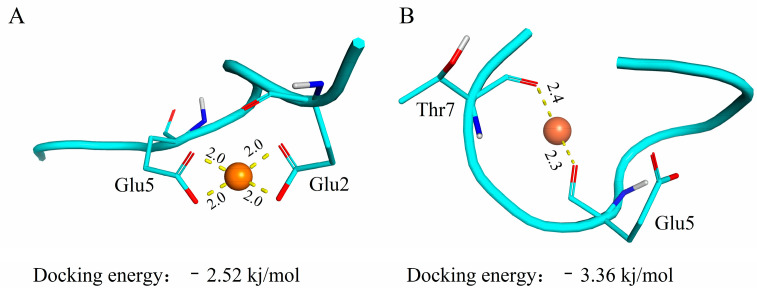
Molecular docking of iron-chelating peptides. (**A**) Interaction between Glu2 and Glu5 in peptide GEHIEESR with iron. (**B**) Interaction between Glu5 and Thr7 in peptide HAVSEGTK with iron. Note: The atoms represented by different colors are as follows: red: O; blue: N; white: H.

Figure 5A,B are the most reliable binding models established by the docking of GEHIEESR and HAVSEGTK with ferrous ions, respectively. It can be seen from the figure that the ferrous ion sites are Glu2, Glu5 and Glu5, Thr7, which also suggests that Glu is one of the main binding sites for ferrous ions. This result is consistent with the results of amino acid composition (Section 3.5). The molecular docking results of the decapeptide zinc chelate prepared by Fan et al. [35] showed that glutamic acid was one of the main amino acids for zinc binding. Subsequently, some studies have proven through molecular docking that the binding of Mytilus edulis osteogenic peptides to calcium ions is mainly due to the contribution of Glu [36,37]. The docking type of ferrous ion with GEHIEESR and HAVSEGTK has electrostatic interactions and metal acceptors. Electrostatic interactions play an important role in the binding of GEHIEESR to ferrous ions. The binding of HAVSEGTK to ferrous ions is as a metal receptor. This result is similar to that of the hydrophobic peptide zinc chelate in oysters [38].

### 3.8. In Vitro Antioxidant Activity

**Figure 6:**
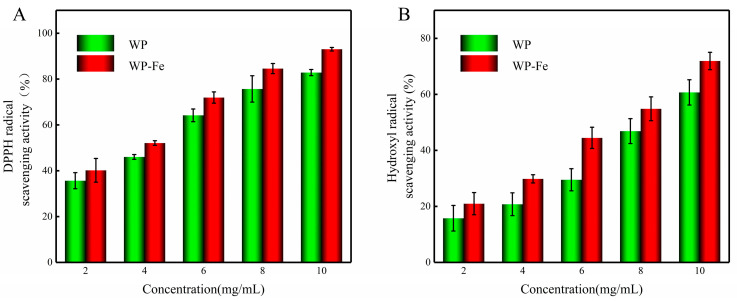
The antioxidant activities of the WP and WP–Fe. (**A**) DPPH radical scavenging activity and (**B**) hydroxyl radical scavenging activity.

Determination of the free radical scavenging rate is one of the more commonly used methods to evaluate the antioxidant activity of active substances. The scavenging ability of WP-Fe for DPPH and hydroxyl radicals was better than that of WP (Figure 6A,B). The IC50 values of the WP-Fe chelate for scavenging DPPH and hydroxyl radicals were 3.72 mg/mL and 7.12 mg/mL, respectively, which were significantly lower than those of the WP (4.43 mg/mL and 8.70 mg/mL). He et al. [16] proved that the antioxidant activity of oat peptide was improved after combining with ferrous ions through in vivo and in vitro experiments. Similarly, Athira et al. [39] proved that the whey protein hydrolyzate after chelating iron still had antioxidant activity through the ABTS* and FRAP experiments. On one hand, the peptide–metal complex system itself has a powerful antioxidant mechanism [32]. On the other hand, the addition of ferrous ions changes the structure of the peptide chain, which may expose more amino acids with antioxidant activity [40]. Therefore, we believe that WP-Fe is a new substance unlike the polypeptides with the dual nutritional effects of antioxidant and iron supplementation.

## 4. Conclusions

In summary, we successfully developed a new iron-fortified food using low molecular weight walnut peptides. Under the conditions of this study, the chelation rate and iron content of the WP-Fe chelate were 71.87 ± 1.60%, 113.11 ± 2.52 mg/g. Various characterization experiments proved that the walnut oligopeptide combined with ferrous ion and formed a honeycomb-like block structure with the increase in hydrodynamic particle size. The identification results of the amino acid composition and peptide chain confirmed that the formation of the WP-Fe chelate mainly depends on the combination of ferrous ions with carboxyl, carbonyl, and amino groups provided by glutamic acid and arginine. In terms of antioxidants, the ability of the WP-Fe chelate to scavenge DPPH and hydroxyl radicals was much stronger than that of WP. Additionally, this study provides a reliable WP-Fe chelate binding model using molecular docking techniques. The WP-Fe chelate synthesized in this study is expected to be used as a dual nutritional supplement. Therefore, it is necessary to carry out the cell test and in vivo absorption test to explore more biological activities.

## Figures and Tables

**Table 1:** Amino acid compositions of the WP and the WP-Fe chelate.

Amino Acids	WP	WP-Fe
Asp	9.66%	9.62%
Thr	3.52%	2.67%
Ser	5.34%	5.69%
Glu	19.86%	22.37%
Gly	4.61%	5.22%
Ala	4.00%	2.99%
Cys	0.74%	0.98%
Val	4.08%	2.64%
Met	3.09%	2.41%
Ile	4.64%	4.61%
Leu	7.08%	3.99%
Tyr	3.19%	2.35%
Phe	4.54%	2.98%
His	2.78%	3.71%
Lys	2.49%	3.39%
Arg	12.93%	19.06%
Pro	7.45%	5.31%

**Table 2:** The sequence description of the WP-Fe peptides.

No.	Peptide Sequence	Length	Mass	Score
1	GEHIEESR	8	955.44	180.7
2	HAVSEGTK	8	827.41	180.18
3	IMELINNVAK	10	1143.63	145.34
4	IPGDIGIKLP	10	1021.62	104.37
5	IDELDSIAPK	10	1099.58	104.2
6	IGGIGTVPVGR	11	1024.60	154.12
7	LQLWDTAGQER	11	1315.65	140.14
8	GVDLIRQGWSR	11	1285.69	117.89
9	INVIGEPIDER	11	1253.66	117.7
10	DAYVGDEAQSK	11	1181.52	117.01
11	IINVIGEPIDER	12	1366.75	163.94
12	DNIQGITKPAIR	12	1324.75	159.79
13	GLLLPSFSNAPR	12	1270.70	109.29
14	AAEVLELAGNAAR	13	1283.68	148.32
15	VSAVNVKQEHSAS	13	1354.68	101.65
16	TGLVIDSGDGVTH	13	1269.62	99.802
17	SMLLTGGSASGGL	13	1149.57	90.657
18	EVVLEKSETVKDT	13	1475.77	85.731
19	ISITDFGGVGDGR	13	1292.64	80.755
20	TTGIVLDSGDGVSH	14	1356.65	134.66
21	YLAGNPHQQQQGGR	14	1552.75	111.31
22	LAAEVLELAGNAAR	14	1396.77	110.38
23	TIHSDHEGGNVSAH	14	1459.64	79.286
24	AVFVDLEPTVIDEVR	15	1700.90	107.83
25	DSGDGVSHTVPIYEGY	16	1694.74	125.75
26	EYLAAEVLELAGNAAR	16	1688.87	109.83
27	LTDQHPEQIVTSEAKGS	17	1838.90	42.813

**Table 3:** Docking profiles of the WP and ferrous ion.

No.	Peptide Sequence	Chelating Sites	Types	Distance
1	GEHIEESR	Glu5; Glu2	M-A; C-C	2.0; 2.0; 2.0; 2.0
2	HAVSEGTK	Glu7; Thr5	M-A	2.3; 2.4
3	IMELINNVAK	Asn6: Asn7	M-A	2.5; 2.4
4	IPGDIGIKLP	Lys8; Leu9	M-A	2.5; 2.4
5	IDELDSIAPK	Asp2; Glu3	M-A; C-C	2.6; 2.6; 2.4
6	IGGIGTVPVGR	Pro8; Val7	M-A	2.4; 2.6
7	LQLWDTAGQER	Thr6; Asp5	M-A; C-C	2.4; 2.6; 2.6
8	GVDLIRQGWSR	Leu4; Asp3	M-A;	2.4; 2.5
9	INVIGEPIDER	Glu10; Asp9	M-A; C-C	2.4; 2.6; 2.6
10	DAYVGDEAQSK	Ala2; Asp1	M-A; C-C	2.4; 2.6; 2.6
11	IINVIGEPIDER	Glu11; Asp10	M-A; C-C	2.4; 2.6; 2.6
12	DNIQGITKPAIR	Asn2; Asp1	M-A; C-C	2.5; 2.6; 2.7
13	GLLLPSFSNAPR	Ala10; Asn9	M-A	2.4; 2.5
14	AAEVLELAGNAAR	Ala11; Asn10	M-A	2.4; 2.5
15	VSAVNVKQEHSAS	Val6; Asn5	M-A	2.4; 2.5
16	TGLVIDSGDGVTH	Ser7; Asp6	M-A; C-C	2.4; 2.6; 2.6
17	SMLLTGGSASGGL	Leu4; Thr5	M-A	2.4; 2.4
18	EVVLEKSETVKDT	Thr13; Asp12	M-A; C-C	2.4; 2.6; 2.6
19	ISITDFGGVGDGR	Thr4; Ile3; Ser2	M-A	2.7; 2.5; 2.6
20	TTGIVLDSGDGVSH	Gly11; Asp10	M-A; C-C	2.4; 2.7; 2.5
21	YLAGNPHQQQQGGR	Pro6; Asn5	M-A	2.4; 2.5
22	LAAEVLELAGNAAR	Ala12; Asn11	M-A	2.4; 2.5
23	TIHSDHEGGNVSAH	His6; Asp5	M-A; C-C	2.4; 2.6; 2.6
24	AVFVDLEPTVIDEVR	Thr9; Pro8; Glu7	M-A	2.4; 2.5; 2.5
25	DSGDGVSHTVPIYEGY	Thr9; His8; Ser7	M-A	2.7; 2.5; 2.7
26	EYLAAEVLELAGNAAR	Leu3; Tyr2	M-A	2.5; 2.6
27	LTDQHPEQIVTSEAKGS	Gln4; Asp3	M-A; C-C	2.4; 2.6; 2.6

Note: The abbreviations of the types are as follows: M-A: metal-acceptor; C-C: charge-charge.

## Data Availability

Data is contained within the article.
